# The orthotic and therapeutic effects following daily community applied functional electrical stimulation in children with unilateral spastic cerebral palsy: a randomised controlled trial

**DOI:** 10.1186/s12887-015-0472-y

**Published:** 2015-10-12

**Authors:** Dayna Pool, Jane Valentine, Natasha Bear, Cyril J. Donnelly, Catherine Elliott, Katherine Stannage

**Affiliations:** Department of Physiotherapy and Paediatric Rehabilitation, Princess Margaret Hospital for Children, Roberts Road, Subiaco, 6008 Australia; Department of Paediatric Rehabilitation, Princess Margaret Hospital for Children, Roberts Road, Subiaco, 6008 Australia; School of Sport Science Exercise and Health, The University of Western Australia, 35 Stirling Highway, Crawley, 6009 Australia; Curtin University of Technology, Faculty of Health Science, Kent Street, Bentley, 6012 Australia; Department of Orthopaedics, Princess Margaret Hospital for Children, Roberts Road, Subiaco, 6008 Australia

**Keywords:** Cerebral palsy, Unilateral spastic cerebral palsy, Spastic hemiplegia, Randomised controlled trial, Ankle kinematics, Temporal-spatial parameters, Orthotic effect, Therapeutic effect, Carry over effect, Functional electrical stimulation

## Abstract

**Background:**

The purpose of this study was to determine the orthotic and therapeutic effects of daily community applied FES to the ankle dorsiflexors in a randomized controlled trial. We hypothesized that children receiving the eight-week FES treatment would demonstrate orthotic and therapeutic effects in gait and spasticity as well as better community mobility and balance skills compared to controls not receiving FES.

**Methods:**

This randomized controlled trial involved 32 children (mean age 10 yrs 3 mo, SD 3 yrs 3 mo; 15 females, 17 males) with unilateral spastic cerebral palsy and a Gross Motor Function Classification System of I or II randomly assigned to a FES treatment group (*n* = 16) or control group (*n* = 16). The treatment group received eight weeks of daily FES (four hours per day, six days per week) and the control group received usual orthotic and therapy treatment. Children were assessed at baseline, post FES treatment (eight weeks) and follow-up (six weeks after post FES treatment). Outcome measures included lower limb gait mechanics, clinical measures of gastrocnemius spasticity and community mobility balance skills.

**Results:**

Participants used the FES for a mean daily use of 6.2 (SD 3.2) hours over the eight-week intervention period. With FES, the treatment group demonstrated a significant (*p* < 0.05) increase in initial contact ankle angle (mean difference 11.9° 95 % CI 6.8° to 17.1°), maximum dorsiflexion ankle angle in swing (mean difference 8.1° 95 % CI 1.8° to 14.4°) normalized time in stance (mean difference 0.27 95 % CI 0.05 to 0.49) and normalized step length (mean difference 0.06 95 % CI 0.003 to 0.126) post treatment compared to the control group. Without FES, the treatment group significantly increased community mobility balance scores at post treatment (mean difference 8.3 units 95 % CI 3.2 to 13.4 units) and at follow-up (mean difference 8.9 units 95 % CI 3.8 to13.9 units) compared to the control group. The treatment group also had significantly reduced gastrocnemius spasticity at post treatment (*p* = 0.038) and at follow-up (dynamic range of motion mean difference 6.9°, 95 % CI 0.4° to 13.6°; *p* = 0.035) compared to the control group.

**Conclusion:**

This study documents an orthotic effect with improvement in lower limb mechanics during gait. Therapeutic effects i.e. without FES were observed in clinical measures of gastrocnemius spasticity, community mobility and balance skills in the treatment group at post treatment and follow-up. This study supports the use of FES applied during daily walking activities to improve gait mechanics as well as to address community mobility issues among children with unilateral spastic cerebral palsy.

**Trial registration:**

Australian New Zealand Clinical Trials Register ACTRN12614000949684. Registered 4 September 2014.

**Electronic supplementary material:**

The online version of this article (doi:10.1186/s12887-015-0472-y) contains supplementary material, which is available to authorized users.

## Background

Cerebral palsy (CP) refers to a group of permanent motor dysfunctions due to non-progressive damage to the developing brain [1]. Unilateral spastic cerebral palsy (USCP) is the most common presentation of CP and children are typically classified as having a Gross Motor Function Classification System (GMFCS) and Winters Gage and Hicks gait classification of I or II [2–4]. This means that despite impairments such as spasticity and muscle contracture particularly at the ankle joint, children remain functionally ambulant. Equinus during gait is a common problem alongside functional issues with balance and community mobility [3, 4].

The neuronal group selection theory provides an essential framework to understanding the balance and community mobility limitations in children with USCP [5]. Based on this framework, children with USCP display primary repertoires of movement that enable functional mobility. However, the combination of impairments usually present in children with USCP may limit the development of secondary repertoires of movement that are essential for movement adaptability [6]. This deficit in movement adaptability restricts activity such as community mobility and balance skills, and may even increase their risk of falls during gait. The expansion of primary and secondary repertoires requires the implementation of the principles of motor learning. Principles of motor learning require treatments to be activity based or task specific that is frequently repeated and challenged in contextually relevant environments [7–9].

Current treatments to improve the gait of children with USCP include pharmacological strategies such as botulinum toxin type A, implemented alongside a range of physiotherapy treatments and/or the prescription of ankle foot orthoses (AFOs) [10]. Although AFOs improve ankle kinematics and temporal-spatial parameters during gait [11, 12], for high functioning children, the external support of an AFO may hinder balance strategies for secondary repertoire expansion as well as impede power generation for effective push off during walking and running [11–13]. Evidence supporting the use of AFOs is mainly focused on the effect it has on body structure and function. It is currently unclear what effect AFOs have with long term use as well as the effect it has on activity and participation [7, 14, 15]. Thus investigation into alternate interventions is warranted.

Functional Electrical Stimulation (FES) has the potential to meet the motor learning needs to expand movement repertoires because it can be implemented frequently during functional tasks such as walking. Muscles are artificially stimulated using an electrical current that is transmitted through electrodes placed over the surface of the skin above the target muscle and nerve [16]. When FES is applied to the ankle dorsiflexors during gait it can act as an orthosis by initiating a muscle contraction to dorsiflex the ankle joint, thus allowing for improved toe clearance during the swing phase of gait (known as the orthotic effect) [17, 18].

In a recent systematic review, the use of lower limb muscle electrical stimulation for improving gait and functional activity was cautiously advocated for children with CP [19]. However included in this review were studies where electrical stimulation was not functionally applied, hence given the overwhelming evidence supporting the need for specificity of treatment, the limited effect on gait and activity is understandable [7, 20, 21]. Since this review, research has emerged with FES applied to the ankle dorsiflexors during the swing phase of gait, and though not randomized controlled trials, results have supported an orthotic effect with improvements in ankle kinematics enabling toe clearance [17, 22, 23]. Determining whether the effects last beyond the treatment period with the removal of FES (known as the therapeutic effect) and whether it improves community mobility and balance skills has not yet been determined and so has been a recommendation for future research in this area [19].

This study aimed to determine the orthotic and therapeutic effects of daily community applied FES to the ankle dorsiflexors in a randomized controlled trial. We hypothesized that children who received the eight-week FES treatment would demonstrate an orthotic effect with improved lower limb kinematics (i.e. elevated dorsiflexion during the swing phase of gait) during the gait cycle compared to controls not receiving FES. Secondly, after the removal of FES, children who were in the FES treatment group would demonstrate a therapeutic effect with improved lower limb kinematics during gait, better community mobility and balance scores and reduced gastrocnemius spasticity compared to controls that did not receive FES.

## Methods

### Study design

The study design was a randomized controlled trial of daily community applied FES to the ankle dorsiflexors in gait compared with usual care (control group).

### Participants

Participants were referred to the study by Physiotherapists and Paediatric Rehabilitation Consultants. Participant inclusion criteria are detailed in Table 1. Participants who had underwent botulinum toxin type A were included, but scheduled to commence the study at three months post injections [24]. Study recruitment took place between June and July 2013 from clinics of the Cerebral Palsy Mobility Service at Princess Margaret Hospital for Children and The Centre for Cerebral Palsy, Perth Australia with the final assessments completed by April 2014. Human ethics approval was obtained from the Human Research Ethics Committees of Princess Margaret Hospital, Perth Australia and The University of Western Australia, Perth Australia. The committee’s recommendations were adhered to and written and informed consent for participation and publication was obtained.

**Table 1:** Inclusion and exclusion criteria

Inclusion criteria	Exclusion criteria
• Passive dorsiflexion range of affected ankle of at least 5°	• History of uncontrolled seizure disorder
• Full passive knee extension bilaterally	• Orthopaedic lower limb surgery on the affected side in the past 12 months
• Dynamic popliteal angle of no more than 45°	• Orthopaedic metal ware at the site of electrical stimulation
• Able to cooperate with assessment procedures	• Botulinum toxin in lower limb in the past 3 months
• Willing to use the Walk Aide® at least 4 hours a day, 6 days a week for 8 weeks	
• GMFCS I or II, unilateral spastic cerebral palsy (with or without dystonia)	
• Aged between 5 and 18	

### Procedure

An initial appointment with the principal investigator (DP) was scheduled to determine FES tolerance and study protocol. Randomization to either the FES or control group was achieved through a coin toss, by an individual uninvolved with the study. Randomization only occurred once two matched participants were enrolled. Matched participants were of the same GMFCS level, and were within two years of age for children aged between five and 10, and within six years for children aged between 11 and 18. This method was applied to improve the homogeneity of each group in terms of age and gross motor function.

Participants were asked not to participate in any new sporting activities during the study and to maintain pre-existing therapy throughout the 14-week study period so that the effects of FES treatment could be determined. The Actigraph® (GT3X, ActiGraph, Penascola, Florida, USA), a triaxial device was used to monitor time spent in moderate to vigorous physical activity (MVPA) [25] because of its potential to confound the overall outcome from FES intervention.

Outcome measures were assessed at baseline, post-treatment (following eight weeks of FES intervention) and follow-up (six weeks after the post-treatment). The presence of an orthotic effect was determined in the between group comparison at post treatment, whilst the treatment group was wearing the FES device during the gait analysis assessment. A therapeutic effect was determined both at post treatment and at follow-up through the examination of between group differences for the community mobility balance measures, spasticity measures, as well as in the gait analysis, but only when the treatment group was not wearing the FES device.

### Outcome measures

This randomized controlled trial of daily community FES assessed outcomes across all domains of the International Classification of Functioning Child and Youth Version. This paper focuses on the results pertaining to the effects of FES on the domains of body structure and function and activity. The primary outcomes were lower limb biomechanics and included ankle kinematics and temporal spatial measures during walking gait cycle and community balance and mobility estimates. The secondary measures were clinical assessments of gastrocnemius spasticity. As this study was conducted within the framework of current clinical care, measures of passive dorsiflexion with the knee extended and popliteal angles were also taken at all assessment time points to ensure no detrimental loss of range of motion over the 14 weeks study period. This was considered to be important particularly in the absence of AFOs. Though this was not an outcome measure, the results may be of interest to clinicians and so are presented as Additional file 1 in this paper.

#### Gait analysis

Two dimensional gait assessment was conducted at The School of Sport Science, Exercise and Health Gait Laboratory at The University of Western Australia. Three Bonita™ cameras (Vicon© Motion Systems Ltd UK) capturing at 100Hz for sagittal (left and right) and coronal (one camera) views were positioned and synchronized to capture video with two AMTI force platforms (1,000Hz). Children were asked to walk at a self- selected walking speed along a 10 m walkway to capture five successful trials i.e. uninterrupted foot strike on force platform. Bright coloured, round stickers were placed on bilateral greater trochanters, anterior superior iliac spine, posterior superior iliac spine, acromion clavicular joint, medial and lateral femoral epicondyles, patella, medial malleoli, lateral malleoli, head of the fifth metatarsal and calcaneus. This allowed identification of specific anatomical landmarks and joint centers during video motion capture. SiliconCoach Pro7 ® (Siliconcoach Ltd, Dunedin, New Zealand), was used for video analysis with initial contact and toe-off identified from the vertical ground reaction force measure from the platforms (>10 N and <10 N respectively). Ankle angle was calculated between the tibia and the foot from the sagittal plane high-speed video (using the markers on the lateral femoral epicondyles, lateral malleoli and head of the fifth metatarsal). Ankle dorsi/plantarflexion angles were calculated at four discrete time points, 1) initial foot contact, 2) maximum dorsiflexion in stance, 3) toe off and 4) maximum dorsiflexion in swing. Temporal-spatial measures included time in stance, step length normalized to height [26], velocity prior to initial foot contact and walking velocity over 5 m. Participants were assessed walking in shoes and in-shoe orthoses (if any) at all assessment time points. Participants in the FES treatment group were assessed both with (to determine the orthotic effect) and without (to determine the therapeutic effect) the FES device at post-treatment. It was not possible to blind the assessor regarding group and time point allocation due to the facial identity and Walk Aide® visibility on the video.

#### Clinical tests

The clinical tests were performed at Princess Margaret Hospital for Children by an experienced physiotherapist (DP) and research assistant, following the outlined protocols at all of the time points. It was not possible to blind the assessors to group or assessment time point allocation.

#### Community mobility and balance skills

The Community Balance and Mobility Scale (CBMS) is a valid and reliable clinical tool that rates performance quality (out of a possible 96 points) of high level community balance and mobility skills in ambulatory patients with neurological impairment [27–29]. It includes items relevant for everyday community mobility such as turning, step-ups, walking and looking, direction changes and picking things up off the ground when walking. The CBMS was chosen because it would not have a ceiling effect for children with a GMFCS level of 1 as used previously by Brien and Sveistrup 2011 [28]. An overall change in score by five points is considered clinically meaningful, reflecting true change in confidence in community mobility and community integration [28].

The 4-Square Step Test (4SST), a valid, reliable and sensitive clinical tool was used to assess dynamic stepping balance and rapid changes in direction [30]. The 4SST measures the time it takes to step over four walking sticks placed in a four square configuration, requiring the participant to step over and clear a height of 2.5 cm in all directions following previously documented protocols [30]. Although this test is not routinely used for children with CP, it was included because of its use to predict falls in people with neurological impairments. A score of 15 seconds or more has been shown to be the cut-off point to identify falls risk in people with neurological impairments [30].

Self reported incidence of toe drag and falls was measured using a questionnaire from our pilot study [23]. The questions asked were “How often do you drag your toes when you are walking?” and “How often do you fall over?” Answers were given on a five point ordinal scale (0–4), with a higher score indicating an increased incidence.

#### Range of motion and spasticity

Goniometry measures of passive and dynamic (Modified Tardieu Scale) ankle dorsiflexion in subtalar neutral (with the knee extended) and popliteal angle in supine were taken by DP following previously documented protocols [23, 31]. A change in angle by 10 degrees was considered clinically meaningful [32]. The Australian Spasticity Assessment Scale (ASAS), a five point ordinal scale was done concurrently to measure spasticity for gastrocnemius and hamstrings because of its proven validity and reliability in documenting spasticity in clinical practice [33]. We considered that a score change of one was clinically meaningful.

### FES intervention

Participants in the FES group received the FES device after the baseline assessment. The Walk Aide® (Innovative Neurotronics, Austin, TX, USA) is a small (8.2 cm × 6.1 cm × 2.1 cm, 87.9 g) device that delivers asymmetrical biphasic surface electrical stimulation (ES) in a synchronized manner to stimulate the motor neurons of the tibialis anterior muscle, which dorsiflexes the ankle during the swing phase of gait. It is attached to the participant’s leg by a cuff that sits just under the knee on the affected side. One electrode was placed on the muscle belly of tibialis anterior and the other on the common peroneal nerve, which innervates tibialis anterior and other ankle dorsiflexors (extensor digitorum longus, peroneus tertius and extensor hallucis longus). During a gait cycle, the Walk Aide® is triggered by an individualized program detecting changes in tibia angle to stimulate ankle dorsiflexion. Specific attention and time was spent by DP to ensure appropriate electrode placement and pulse width settings for accurate ankle dorsiflexion without excessive and unwanted movements of the foot. The set up procedure followed that described in our pilot study [23]. Participants and parents were supported so that they were confident and independent with the FES device, ensuring balanced dorsiflexion (no excessive subtalar eversion) with every use. Weekly to fortnightly visits at home or school were necessary to support daily FES use. This included training parents, teachers and education assistants on the use of the device as well as conducting classroom talks so that the participant’s peers were aware of what the device was and why it was being worn. These visits also enabled electrode placement and integrity checks and inspection for any adverse events.

Participants were asked to use the FES device for at least four hours per day, six days per week during the eight-week treatment period. This was monitored through the usage log on the device itself. To enable participants an opportunity to accommodate to the device, they were asked to build up gradually to the required dosage over the first week.

Participants in the FES intervention group did not wear their AFO throughout the study period. They were all provided with customized in-shoe orthoses at the commencement of the study to support foot posture and account for leg length discrepancies particularly in the absence of AFOs. Participants in the control group were asked to continue with their usual orthotic protocol.

### Statistical analysis

Based on effect sizes observed in our pilot study of FES use [23], a one tailed alpha of 0.05 and power of 80 % power analysis suggested that each group required at least 15 participants per group to detect a clinically meaningful change in functional muscle strength (by six heel raises).

Normality was established for all clinical and gait measures through examining distributional plots, Q-plots and the Shapiro-Wilk test. Means and standard deviations were reported for each group for each phase. Determining between group differences was the main focus and this was examined using repeated measures ANCOVA (using the baseline as the covariate) to account for the correlation between repeated measures over time. Tukey’s post-hoc analysis was applied if a significant main effect for group and time or an interaction of these was found enabling appropriate adjustments for the multiple comparisons and calculation of mean differences and 95 % confidence intervals. To better understand the significance of the statistically significant comparisons for the gait data, effect sizes were also determined by using Cohen’s *d* calculation with a value of 0.8 considered a large effect, 0.5 to be a medium and 0.2 to be a small effect [34]. Assumptions for the ANCOVA were examined and met. Actigraphy® was only measured at baseline and post treatment and so was examined using an independent t-test. The Mann–Whitney U test was used to determine between group differences for ordinal scales of ASAS and self-reported toe-drag and falls with medians and interquartile ranges reported for each group in each phase. Statistical significance was accepted as *p <* 0.05. All statistical analyses were performed using STATA version 12.1 (TestCorp, Texas).

## Results

Thirty-two children, mean age 10 y 8 mo (SD 3y 3mo) with USCP GMFCS I or II, were recruited for the study. Baseline participant characteristics are shown in Table 2. All participants had spasticity in the lower limb, three participants had mixed tone with spasticity and dystonia (as indicated by the Hypertonia Assessment Tool) [35]. All participants who attended the initial appointment completed the study. There was no missing clinical data in the study, with all 32 participants assessed at all three-time points in their original group allocation.

**Table 2:** Baseline characteristics

	FES (*n* = 16)	Control (*n* = 16)	*p* value
Weight (kg)	38.5 (15.2)	37.4 (15.9)	
Gender	Male: 9	Male: 8	
	Female: 7	Female: 8	
Side of hemiplegia	Right: 11	Right: 12	
	Left: 5	Left: 4	
GMFCS	I: 10	I: 10	
	II: 6	II: 6	
Age	10y 11mo (3y 10mo)	10y 5mo (2y 8mo)	
AFO	10	11	
Winters Gage and Hicks	I: 0	I: 1	
	II: 16	II: 15	
Clinical measures			
Gastrocnemius ASAS	2 (1.5-3)	2 (1.5-3)	1.00^a^
Passive Dorsiflexion (°) (knee extended)	11.94 (5.87)	10.5 (5.54)	0.482^b^

^a^Mann Whitney U test; ^b^t test; *GMFCS* Gross Motor Function Classification System, *AFO* Ankle Foot orthoses, *ASAS* Australian Spasticity Assessment Scale

There were no clinically significant differences in the primary outcome measures between the groups at baseline (baseline values provided in Tables 3 and 4). Actigraphy® data was returned in all but one participant at baseline. There were no significant differences in MVPA between the groups at baseline (*p* = 0.428) and post treatment (*p* = 0.931).

**Table 3:** Mean (SD) of groups and corresponding mean difference between groups (95 % CI) reported for spasticity and activity clinical measures at baseline (A), post treatment (B) and follow-up (C)

		FES	Control	Mean difference (95 % CI)	Between group *p* value
ASAS gastrocnemius^a^	A	2 (1.5-3)	2 (1.5-3)		
	B	1 (0.5-2)	2 (2–2)	-	*p* = 0.038^b^
	C	2 (0.5-3)	2 (2–3)	-	*p* = 0.090
Dynamic dorsiflexion ROM (°)	A	0 (10.7)	1.1 (8.8)		
	B	5.9 (9.4)	1.9 (7.1)	4.7 (−1.9 to 11.3)	*p* = 0.245
	C	4.5 (9.8)	−1.8 (10.9)	6.9 (0.4 to 13.6)	*p* = 0.035^b^
4SST (seconds)	A	10.9 (2.8)	10.6 (3.3)		
	B	9.0 (2.6)	9.6 (2.1)	−0.1 (−0.2 to 0.03)	*p =* 0.182
	C	8.5 (2.8)	9.1 (2.6)	−0.1 (−0.3 to 0.03)	*p* = 0.160
Self-report toe drag^a^	A	2 (1.5-4)	4 (2–5)		
	B	2 (1–3)	4 (2.5-5)	-	*p* = 0.002^b^
	C	2 (1–3.5)	4 (2–4)	-	*p* = 0.069
CMBS (score out of 96)	A	56.4 (14.8)	53.5 (16.5)		
	B	67.7 (12.8)	56.9 (16.9)	8.3 (3.2 to 13.4)	*p* < 0.001^b^
	C	70.4 (11.3)	58.9 (16.2)	8.9 (3.8 to 13.9)	*p* < 0.001^b^
Self-report falls^a^	A	2 (1–3)	3 (2–4)		
	B	2 (1–2.5)	2 (2–3)	-	*p =* 0.089
	C	1.5 (1–2)	2.5 (2–3.5)	-	*p* = 0.011^b^

*ASAS* Australian Spasticity Assessment Scale, *ROM* Range of Motion, *4SST* Four Square Step Test, ^a^Mann Whitney U tests with reported medians and IQR; ^b^Significant difference between the groups *p* < 0.05; − Calculation and test not indicated; *CBMS* Community Balance Mobility Scale

**Table 4:** Mean (SD) of groups and corresponding mean difference between groups (95 % CI) reported for gait kinematics and temporal-spatial parameters at baseline (A), post treatment (B) and follow-up (C)

	Group							Mean Difference between groups (95 % CI)		
	A		B			C		B	B	C
Outcome	Rx	Con	Rx	Rx	Con	Rx	Con	Rx FES - Con	Rx No FES - Con	Rx No FES - Con
	No FES		FES	No FES		No FES				
Initial contact ankle angle (°)	−7.8	−5.5	3.2	−5.1	−7.6	−6.1	−6.6	11.9	-	-
	(6.7)	(8.2)	(6.1)	(7.9)	(8.2)	(7.8)	(8.1)	(6.8 to 17.1)*		
Max. DF stance (°)	12.9	13.8	15	14.8	12.9	13	12.0	-	-	-
	(5.7)	(7.6)	(4.5)	(4.4)	(6.6)	(4.9)	(7.3)			
Max. DF swing (°)	−6.1	−4.6	3.4	−3.3	−3.8	−2.1	−4	8.1	-	-
	(9.5)	(9.5)	(9.6)	(6.9)	(8.0)	(8.4)	(7.6)	(1.8 to 14.4)*		
Toe-off ankle angle (°)	−7.9	−14.2	−11.2	−15.7	−16.2	−16.8	−16.2	-	-	-
	(12.5)	(13.1)	(9.9)	(8.9)	(8.8)	(9.2)	(12.1)			
Time in stance^a^	2.1	2.2	2.3	2.3	2.1	2.2	2.1	0.27	0.23	-
	(0.3)	(0.3)	(0.3)	(0.3)	(0.2)	(0.2)	(0.3)	(0.05 to 0.49)*	(−0.001 to 0.47)*	
Step length – hemi^a^	0.4	0.4	0.5	0.5	0.4	0.5	0.5	0.06	-	-
	(0.1)	(0.1)	(0.1)	(0.1)	(0.1)	(0.1)	(0.1)	(0.003 to 0.126)*		
Step length- non hemi^a^	0.5	0.5	0.5	0.5	0.5	0.5	0.5	-	-	-
	(0.1)	(0.1)	(0.1)	(0.1)	(0.1)	(0.1)	(0.1)			
Ankle velocity before initial contact^a^	0.4	0.5	0.5	0.5	0.5	0.5	0.5	-	-	-
	(0.1)	(0.2)	(0.1)	(0.2)	(0.1)	(0.1)	(0.1)			
Walking velocity (m/s)	1.2	1.2	1.2	1.1	1.2	1.2	1.2	0.04	−0.04	−0.04
	(0.2)	(0.2)	(0.2)	(0.2)	(0.2)	(0.2)	(0.2)	(0.09 to −0.12)	(−0.10 to 0.11)	(−0.09 to 0.02)

*Rx* treatment group, *Con* control group, *FES* Functional Electrical Stimulation, −, Calculation and test not indicated; ^a^Values reported are normalized dimensionless values; *p <0.05

All participants had the FES device set with a frequency of 33Hz with pulse width ranging from 25 to 100 μs. Participants used the FES for a mean daily use of 6.2 (SD 3.2) hours over the eight-week intervention period. There were no reported unintended effects or adverse events.

### Gait measures

At the post treatment assessment, the groups were significantly different (with small to medium effect sizes) when the treatment group was wearing the FES device (Table 4). With the FES device on at post treatment, the treatment group had an increased ankle angle at initial contact (mean difference 11.9°, 95 % CI 6.8° to 17.1°; *p* < 0.001; *d* = 0.6), increased ankle angle in maximum dorsiflexion in swing (mean difference 8.1°, 95 % CI 1.8 to 14.4°; *p* = 0.007; *d* = 0.4), increased normalized time in stance (mean difference 0.27, 95 % CI 0.05 to 0.49; *p* = 0.011; *d* = 0.4) and increased normalized step length on the affected side (mean difference 0.06, 95 % CI 0.003 to 0.126; *p* = 0.035; *d* = 0.4) when compared to the control group. Without the FES device on at post treatment, the treatment group continued to demonstrate increased normalized time in stance (mean difference 0.23, 95 % CI −0.001 to 0.47; *p* = 0.050; *d* = 0.4) when compared to the control group and this was considered a small/medium effect size. There were no other significant differences between the groups for the remaining ankle kinematic and temporal spatial gait measures at post treatment and at follow-up.

### Activity clinical measures

The CBMS scores were significantly different between the groups with the treatment group demonstrating higher scores both at post treatment, (mean difference 8.3 units, 95 % CI 3.2 to 13.4 units; *p* < 0.001) and at follow-up (mean difference 8.9 units, CI 3.8 to 13.9 units; *p* < 0.001). After the FES treatment, the treatment group had a significant reduction in the incidence of self-reported toe drag (*p* = 0.002) and a significant reduction in self-reported falls at follow-up (*p* = 0.022) when compared to the control group.

### Spasticity and range of movement

There were significant differences between the groups for gastrocnemius spasticity with the median score in the treatment group decreasing from ASAS 2 at baseline to ASAS 1 post treatment (*p* = 0.038). The groups were also significantly different at follow-up, with the treatment group having increased dynamic ankle dorsiflexion range (mean difference 6.9°, 95 % CI 0.4° to 13.6°; *p* = 0.035). There were no significant differences between the groups for passive dorsiflexion and popliteal angle range of movement post treatment and at follow-up (Additional file 1). Notably, there was no mean loss of ankle or knee range of motion at both assessment time points in the treatment group.

## Discussion

Supporting our first hypothesis, this study documents evidence of an FES orthotic effect in gait with improvements in ankle kinematics to enable toe clearance when walking. The improvement in ankle kinematics further strengthens the current literature supporting the use of FES to the ankle dorsiflexors in children with USCP, to increase the ankle angle in swing to functionally reduce toe drag when walking [18, 22, 36, 37]. The improvement in the time spent in stance on the affected leg provides further evidence that FES in swing can also affect some stance phase features. Once again this strengthens previous results where this effect has also been reported, but only in three children with CP [18]. Hence FES seems to offer some limited but similar features to AFOs, in terms of its effectiveness in improving ankle kinematics, time spent in stance and step length [11–13]. For children who do not require the stance phase knee and hip control that is offered by AFOs, clinicians may consider the implementation of FES for children with USCP that exhibit equinus gait patterns.

Supporting our second hypothesis, after eight-weeks of FES, and with the removal of the FES device, participants in the treatment group demonstrated a therapeutic effect with significantly better CBMS scores, reduced gastrocnemius spasticity and self-reported toe drag compared to the control group. There has been limited evidence supporting the therapeutic effect in CP and this has largely been attributed to variable intervention parameters with different length and setting of intervention, different target muscles for stimulation and underpowered sample sizes to detect significant differences [37–41]. However, the compelling evidence supporting the therapeutic effect in the adult post stroke population, where FES is also used for drop foot has been largely attributed to the application of FES in functional contexts [42]. Therefore the results from the current study support the implementation of daily community applied FES, as this appears to be a necessary component particularly if the goal is to achieve a therapeutic effect.

The mechanism for the therapeutic effect observed at post treatment is unclear. We reason that the reduced gastrocnemius spasticity, improvement in time in stance and community mobility and balance skills reflect more co-ordinated muscle activation at the ankle joint. Referred to as muscle co-contraction due to impaired reciprocal inhibition that is often observed at the ankle during gait, [43] can be used as a strategy to improve joint stability [44]. However it may also be functionally detrimental by impairing co-ordinated muscle activation consequently impacting balance control to result in asymmetrical gait patterns [45]. Stimulation to the ankle dorsiflexors may address problems with reciprocal inhibition due to the repetitive nature of the intervention by moving the ankle in and out of dorsiflexion with each step [18, 43]. In effect, this would enable more balanced muscle function at the ankle, improving stability thus accounting for the improvement in community mobility and balance scores.

The continued therapeutic effect in community mobility and balance skills noted at follow-up supports our pilot study results [23]. These changes provide some evidence to suggest the role of motor learning with the development of secondary repertoires of movement. This could be because the participants’ ambulation needs were challenged as they no longer had the orthotic benefits of FES or an AFO. Further work to substantiate the possibility of neuroplastic changes is therefore warranted in future studies. The evidence for supporting the therapeutic effect particularly regarding community mobility and balance skills is functionally important as it means that these changes are possible with minimal therapy face time, a significant consideration in community clinical practice.

There were no ankle kinematic gait therapeutic effects, suggesting that orthotic effects, as with AFOs, are use-dependent. We speculate that the absence of ankle kinematic therapeutic effects could be attributable to inadequate length or dosage of treatment. However, it could also be attributable to inadequate elicitation of the central nervous system from the FES settings as higher frequencies were not available, whilst higher pulse widths only resulted in discomfort and unwanted excessive movements into ankle eversion [46]. Contrary to previous reports, there were no significant improvements in passive ankle dorsiflexion range of motion [47]. It is worth noting that there was no significant loss in range of motion either. This is an important finding because it demonstrates that the removal of an AFO for a short period of FES will not detrimentally affect ankle range of motion for children in this age group. However, it should be noted that participants in this study did not have high levels of spasticity at baseline, hence these results are limited to children with a Winters Gage and Hicks classification of I and II with a gastrocnemius ASAS of no more than three.

Literature supporting the use of AFOs to maintain or even improve ankle range of motion has methodological limitations such as difficulties with standardization of materials and limitations in the outcome measures used. However, it continues to be acceptable in current clinical practice because it is coupled with clinical expertise and assessment [7, 12]. Certainly wearing AFOs or even using FES does not replace the need for vigorous range of movement monitoring, pharmacological or orthopaedic interventions. Individual assessments continue to be necessary when considering and applying FES for the orthotic and therapeutic effects. Specifically, clinicians will need to evaluate the effectiveness of dorsiflexion stimulation without exacerbating any pre-existing foot deformities as well as to ensure the lower limb biomechanical requirements are met before applying FES in gait i.e. able to meet the inclusion criteria specified for this study. The role of community therapy is highlighted here to ensure that FES is used appropriately at home, school and community.

Whilst the results do support both the orthotic and therapeutic effects of FES in a randomised controlled trial, there are some study limitations to note. Gait analysis was performed using two-dimensional video for easy replication in the community. The reliability of using software for sagittal plane measurements has been established [48] and our results match previous ankle kinematic measures obtained from three-dimensional analysis [22]. This procedure was also enhanced with force platforms to accurately determine significant gait events. However, three-dimensional analyses would have offered gait kinetic information. Also, it was not possible to blind the assessor either during the clinical assessments or during the gait video analysis due to observable facial identify and the presence of a Walk Aide® visibly attached to the leg. To our knowledge, there are no valid measures of toe drag and falls for this population. We therefore developed our own questionnaire for this study, which was used in our pilot study, but has not been validated. There was some missing Actigraph® data and this may have influenced the results. Due to limited number of Actigraph® devices available, follow-up assessments were not possible. Inclusion of this data for the follow-up assessment time point would have strengthened the study to confirm that the therapeutic effect was due to the residual effect of FES and not due to increased levels of MVPA. Another limitation is that although a statistically significant difference in dynamic ankle dorsiflexion range was determined between groups, the mean change did not exceed the variability in measurement at the joint [32]. Finally, many variables were explored here over several time points and this may be a limitation because of the potential for Type I error. The strength of this study however is the high compliance, with no missing data or drop-outs. This reflects the acceptability of the intervention as well as the efficacy and potential for this intervention to be implemented in community clinical practice.

## Conclusion

Short-term daily community FES is an effective activity based treatment with both orthotic and therapeutic effects. The improvements in community mobility and balance skills and spasticity are evident for up to six weeks post treatment. This suggests that FES applied during everyday walking activities is a viable treatment option for children with USCP and equinus gait patterns.

## Consent

Written informed consent was obtained from the patient for publication of this manuscript and any accompanying images. A copy of the written consent is available for review by the Editor of this journal.

## Additional file

Additional file 1:
**Mean (SD) of groups and corresponding mean difference between groups (95 % CI) reported for passive range of motion and spasticity clinical measures at baseline (A), post treatment (B) and follow-up (C).** (DOC 77 kb)

## Abbreviations

FES: Functional Electrical stimulation
SD: Standard Deviation
CI: Confidence Interval
CP: Cerebral palsy
GMFCS: Gross Motor Function Classification System
USCP: Unilateral spastic cerebral palsy
AFO: Ankle foot orthosis
MVPA: Moderate to vigorous physical activity
CBMS: Community balance mobility scale
4SST: Four square step test
ASAS: Australian Spasticity Assessment Scale

## References

[1] Rosenbaum P, Paneth N, Leviton A, Goldstein M, Bax M (2007). The definition and classification of cerebral palsy contents foreword historical perspective definition and classification document. Dev Med Child Neurol..

[2] Beckung E, Carlsson G, Carlsdotter S, Uvebrant P (2007). The natural history of gross motor development in children with cerebral palsy aged 1 to 15 years. Dev Med Child Neurol.

[3] Winters TF, Gage JR, Hicks R (1987). Gait patterns in spastic hemiplegia in children and young adults. J Bone Jt Surg.

[4] Palisano R, Rosenbaum P, Walter S, Russell D, Wood E, Galuppi B (1997). Development and reliability of a system to classify gross motor function in children with cerebral palsy. Dev Med Child Neurol..

[5] Hadders-Algra M (2000). The neuronal group selection theory: promising principles for understanding and treating developmental motor disorders. Dev Med Child Neurol.

[6] Hadders-Algra M (2010). Variation and variability: key words in human motor development. Phys Ther.

[7] Novak I, McIntyre S, Morgan C, Campbell L, Dark L, Morton N (2013). A systematic review of interventions for children with cerebral palsy: state of the evidence. Dev Med Child Neurol.

[8] Eyre JA (2007). Corticospinal tract development and its plasticity after perinatal injury. Neurosci Biobehav Rev..

[9] Johnston MV (2009). Plasticity in the developing brain: implications for rehabilitation. Dev Disabil Res Rev.

[10] Love SC, Novak I, Kentish M, Desloovere K, Heinen F, Molenaers G (2010). Botulinum toxin assessment, intervention and after-care for lower limb spasticity in children with cerebral palsy: international consensus statement. Eur J Neurol.

[11] Romkes J, Brunner R (2002). Comparison of a dynamic and a hinged ankle-foot orthosis by gait analysis in patients with hemiplegic cerebral palsy. Gait Posture.

[12] Buckon CE, Thomas SS, Jakobson-Huston S, Sussman M, Aiona M (2001). Comparison of three ankle-foot orthosis configurations for children with spastic hemiplegia. Dev Med Child Neurol.

[13] Desloovere K, Molenaers G, Van Gestel L, Huenaerts C, Van Campenhout A, Callewaert B (2006). How can push-off be preserved during use of an ankle foot orthosis in children with hemiplegia? A prospective controlled study. Gait Posture.

[14] Autti-Ramo I, Suoranta J, Anttila H, Malmivaara A, Makela M (2006). Effectiveness of upper and lower limb casting and orthoses in children with cerebral palsy. Am J Phys Med Rehabil.

[15] Figueiredo EM, Ferreira GB, Maia Moreira RC, Kirkwood RN, Fetters L (2008). Efficacy of ankle-foot orthoses on gait of children with cerebral palsy: systematic review of literature. Pediatr Phys Ther.

[16] Reed B (1997). The physiology of neuromuscular electrical stimulation. Pediatr Phys Ther..

[17] Prosser LA, Curatalo LA, Alter KE, Damiano DL (2012). Acceptability and potential effectiveness of a foot drop stimulator in children and adolescents with cerebral palsy. Dev Med Child Neurol.

[18] Postans NJ, Granat MH (2005). Effect of functional electrical stimulation applied during walking, on gait in spastic cerebral palsy. Dev Med Child Neurol..

[19] Cauraugh JH, Naik SK, Hsu WH, Coombes SA, Holt KG (2010). Children with cerebral palsy: a systematic review and meta-analysis on gait and electrical stimulation. Clin Rehabil.

[20] Franki I, Desloovere K, De Cat J, Feys H, Molenaers G, Calders P (2012). The evidence-base for basic physical therapy techniques targeting lower limb function in children with cerebral palsy: a systematic review using the International Classification of Functioning, Disability and Health as a conceptual framework. J Rehabil Med.

[21] Alon G (2006). Electrical stimulation in cerebral palsy: are we asking clinically relevant questions?. Dev Med Child Neurol.

[22] Meilahn JR (2013). Tolerability and effectiveness of a neuroprosthesis for the treatment of footdrop in pediatric patients with hemiparetic cerebral palsy. PMR.

[23] Pool D, Blackmore AM, Bear N, Valentine J (2014). Effects of short-term daily community walk aide use on children with unilateral spastic cerebral palsy. Pediatr Phys Ther.

[24] Graham K, Selber P (2003). Musculoskeletal aspects of cerebral palsy. J Bone Joint Surg Br.

[25] Freedson P, Melanson E, Sirard J (1998). Calibration of the Computer Science and Applications, Inc. accelerometer. Med Sci Sport Exerc..

[26] Hof AL (1996). Scaling gait data to body size. Gait Posture..

[27] Howe JA, Inness EL, Venturini A, Williams JI, Verrier MC (2006). The Community Balance and Mobility Scale-a balance measure for individuals with traumatic brain injury. Clin Rehabil.

[28] Brien M, Sveistrup H (2011). An intensive virtual reality program improves functional balance and mobility of adolescents with cerebral palsy. Pediatr Phys Ther.

[29] Wright FV, Ryan J, Brewer K (2010). Reliability of the Community Balance and Mobility Scale (CB&M) in high-functioning school-aged children and adolescents who have an acquired brain injury. Brain Inj.

[30] Dite W, Temple VA (2002). A clinical test of stepping and change of direction to identify multiple falling older adults. Arch Phys Med Rehabil.

[31] Boyd R, Graham HK (1999). Objective measurement of clinical findings in the use of botulinum toxin type A for the management of children with cerebral palsy. Eur J Neurol.

[32] McDowell BC, Hewitt V, Nurse A, Weston TBR (2000). The variability of goniometric measurements in ambulatory children with spastic cerebral palsy. Gait Posture.

[33] Love S, Gibson N, Cole J, Williams N, Blair E. The reliability of the Australian Spasticity Assessment Scale (Abstract). Dev Med Child Neurol. 2008;50(Suppl 113):4-17. 10.1111/dmcn.13000 26762706

[34] Spink MJ, Fotoohabadi MR, Menz HB (2010). Foot and ankle strength assessment using hand-held dynamometry: Reliability and age-related differences. Gerontology.

[35] Jethwa A, Mink J, Macarthur C, Knights S, Fehlings T, Fehlings D (2009). Development of the Hypertonia Assessment Tool (HAT): a discriminative tool for hypertonia in children. Dev Med Child Neurol.

[36] Pierce S, Orlin M, Lauer R, Johnston T, Smith B, McCarthy J (2004). Comparison of percutaneous and surface functional electrical stimulation during gait in a child with hemiplegic cerebral palsy. Am J Phys Med Rehabil.

[37] Durham S, Eve L, Stevens C, Ewins D (2004). Effect of Functional Electrical Stimulation on asymmetries in gait of children with hemiplegic cerebral palsy. Physiotherapy.

[38] Van der Linden ML, Hazlewood ME, Hillman SJ, Robb JE (2008). Functional electrical stimulation to the dorsiflexors and quadriceps in children with cerebral palsy. Pediatr Phys Ther.

[39] Seifart A, Unger M, Burger M (2010). Functional Electrical Stimulation to lower limb muscles after botox in children with cerebral palsy. Pediatr Phys Ther..

[40] Comeaux P, Patterson N, Rubin M, Meiner R (1997). Effect of neuromucular electrical stimulation during gait in children with cerebral palsy. Pediatr Phys Ther..

[41] Carmick J (1993). Clinical use of neuromuscular electrical stimulation for children with cerebral palsy, Part 1: Lower extremity. Phys Ther.

[42] Rushton DN (2003). Functional Electrical Stimulation and rehabilitation — an hypothesis. Med Eng Phys.

[43] Leonard CT, Sandholdt DY, Mcmillan JA, Queen S (2006). Short- and Long-latency contributions to recicpocal inhibition during various levels of muscle contraction of individuals with cerebral palsy. J Child Neurol..

[44] Gross R, Leboeuf F, Hardouin JB, Lempereur M, Perrouin-Verbe B, Remy-Neris O (2013). The influence of gait speed on co-activation in unilateral spastic cerebral palsy children. Clin Biomech.

[45] Patterson KK, Gage WH, Brooks D, Black SE, McIlroy WE (2010). Evaluation of gait symmetry after stroke: a comparison of current methods and recommendations for standardization. Gait Posture.

[46] Bergquist AJ, Clair JM, Lagerquist O, Mang CS, Okuma Y, Collins DF (2011). Neuromuscular electrical stimulation: implications of the electrically evoked sensory volley. Eur J Appl Physiol.

[47] Hazlewood ME, Brown JK, Rowe PJ, Salter P (1994). The use of therapeutic electrical stimulation in the treatment of hemiplegic cerebral palsy. Dev Med Child Neurol..

[48] Grunt S, van Kampen PJ, van der Krogt MM, Brehm M-A, Doorenbosch CM, Becher JG (2010). Reproducibility and validity of video screen measurements of gait in children with spastic cerebral palsy. Gait Posture.

