# Supplementary material for: The orthotic and therapeutic effects following daily community applied functional electrical stimulation in children with unilateral spastic cerebral palsy: a randomised controlled trial
Source: BMC Pediatr. 2015 Oct 12;15:154. doi: 10.1186/s12887-015-0472-y (PMC4603297; doi:10.1186/s12887-015-0472-y)
Supplement: Additional file 1: — Mean (SD) of groups and corresponding mean difference between groups (95 % CI) reported for passive range of motion and spasticity clinical measures at baseline (A), post treatment (B) and follow-up (C). (DOC 77 kb) [file 12887_2015_472_MOESM1_ESM.doc]

Appendix A: Mean (SD) of groups and corresponding mean difference between groups (95% CI) reported for passive range of motion and spasticity clinical measures at baseline (A), post treatment (B) and follow-up (C)

|  |  | FES | Control | Mean difference (95% CI) | Between group *p* value |
| --- | --- | --- | --- | --- | --- |
| Ankle dorsiflexion ROM (°) | A  B  C | 11.9 (5.9)  11.7 (6.5)  12 (5.2) | 10.5 (5.5)  12.6 (6.3)  9.6 (6.5) | -1.9 (-6.1 to 2.2)  1.3 (-2.9 to 5.5) | *p* = 0.608  *p* = 0.846 |
| Popliteal angle (°) | A  B  C | 37.5 (12.2)  34.3 (14.5)  37.1 (13.8) | 42.7 (11.8)  39.2 (11.1)  40.5 (11.9) | -  - | -  - |
| Dynamic popliteal angle (°) | A  B  C | 45.5 (12.9)  38.4 (17.8)  45.4 (17.5) | 48.1 (9.9)  45.3 (16.1)  54.7 (10.2) | -  - | -  - |
| ASAS hamstrings ^a^ | A  B  C | 2 (0.5-2)  1.5 (0-2)  2 (0-2) | 0.5 (0-2)  2 (0-2)  2 (2-2) | -  - | *p*=0.103  *p*=0.070 |

ROM, Range of Motion; ASAS, Australian Spasticity Assessment Scale, ^a^ Mann Whitney U tests with reported medians and IQR
